# Neural bases of gaze and emotion processing in children with autism spectrum disorders

**DOI:** 10.1002/brb3.6

**Published:** 2011-09

**Authors:** Mari S Davies, Mirella Dapretto, Marian Sigman, Leigh Sepeta, Susan Y Bookheimer

**Affiliations:** 1 Department of Psychology, University of California Los Angeles90095, USA; 2 Ahmanson-Lovelace Brain Mapping Center, Semel Institute for Neuroscience and Human Behavior, University of California Los Angeles90095, USA; 3 Department of Psychiatry and Biobehavioral Sciences, David Geffen School of Medicine, University of California Los Angeles90095, USA; 4 FPR-UCLA Center for Culture, Brain, and Development, University of California Los Angeles90095, USA

**Keywords:** Autism, facial expression, functional magnetic resonance imaging, gaze, developmental neuroimaging

## Abstract

Abnormal eye contact is a core symptom of autism spectrum disorders (ASD), though little is understood of the neural bases of gaze processing in ASD. Competing hypotheses suggest that individuals with ASD avoid eye contact due to the anxiety-provoking nature of direct eye gaze or that eye-gaze cues hold less interest or significance to children with ASD. The current study examined the effects of gaze direction on neural processing of emotional faces in typically developing (TD) children and those with ASD. While undergoing functional magnetic resonance imaging (fMRI), 16 high-functioning children and adolescents with ASD and 16 TD controls viewed a series of faces depicting emotional expressions with either direct or averted gaze. Children in both groups showed significant activity in visual-processing regions for both direct and averted gaze trials. However, there was a significant group by gaze interaction such that only TD children showed reliably greater activity in ventrolateral prefrontal cortex for direct versus averted gaze. The ASD group showed no difference between direct and averted gaze in response to faces conveying negative emotions. These results highlight the key role of eye gaze in signaling communicative intent and suggest altered processing of the emotional significance of direct gaze in children with ASD.

## Introduction

Processing and interpreting eye gaze cues is crucial for social development. Neonates orient preferentially to eyes, young infants find direct eye contact physiologically soothing, and by 5 months of age, infants shift their own visual attention reflexively based on others’ eye gaze direction alone (Johnson et al. 1991; Hains and Muir 1996; Mondloch et al. 1999; Farroni et al. 2004). Such early preferences make evolutionary sense, given that they allow for the development of critical skills such as following, sharing, and responding to the attention of others, and contribute to early language development (Mundy et al. 1987; Charman et al. 1997; Carpenter et al. 1998). Gaze cues convey rich social information, and, over time, teach contingencies between the emotions and intentions of others and actions and events in the world.

The brain appears to be especially sensitive to gaze direction in processing features of the face (Wicker et al. 1998; Kawashima et al. 1999; Hoffman and Haxby 2000). Behavioral and functional magnetic resonance imaging (fMRI) studies have found that direction of eye gaze provokes an automatic, reflexive orienting of covert spatial attention (e.g., Friesen and Kingstone 1998), and affects responses in brain structures such as the amygdala and ventral striatum, involved in processing emotional signals such as threat or reward, during the observation of expressive faces (Kawashima et al. 1999; George et al. 2001; Kampe et al. 2001; Adams et al. 2003). One early fMRI study revealed the importance of temporal regions in processing shifts of eye gaze (Puce et al. 1998), and a related study established that activity in these areas is sensitive to context and the perceived intentions of others (Pelphrey et al. 2003). Such work illustrates that gaze is important for decoding important aspects of our social environments, including cues about others’ mental states. Furthermore, this decoding likely works in concert with interpreting emotional cues. For instance, the meaning and social significance of a negative emotional expression differs markedly if it is directed toward or away from the receiver, each indicating a very different communicative intention. To date, however, little research has focused on the neural bases of eye gaze processing within the communicative context of conveying emotional states or intentions.

Given that gaze cues impart critical information regarding others’ feelings and intentions, it is not surprising that abnormalities in gaze processing are prevalent among individuals with autism, who display severe impairments in social functioning and understanding. Reduced attention to faces, and specifically the eyes, in the first year of life is associated with the development of autism (Osterling et al. 2002). Toddlers who develop autism often show profoundly delayed gaze following and joint attention, which has been found to predict subsequent language delays (Sigman et al. 1986; Mundy et al. 1987). Reduced or poorly modulated eye contact typically continues into childhood and beyond. When adults with autism do attend to faces, they have been found to fixate less on the eyes, unless explicitly instructed to do so (Pelphrey et al. 2002). Such abnormalities may also underlie characteristic impairments in recognizing and interpreting emotions, which are disproportionately conveyed by the eyes. Work on gaze fixation behavior of babies with autism has been difficult to attain, but one study found that when cued to pay attention to the eyes, 2-year olds with autism will orient their attention in response to averted gazes (Chawarska et al. 2003). However, unlike typically developing (TD) toddlers who show enhanced response to facial gaze direction, toddlers with autism respond equally well to directional, nonsocial symbols.

Why eye cues appear not to be as salient for individuals with autism, and how this relates to the abnormal development of other neural systems in childhood, is largely unknown. Neuroimaging studies have recently begun to address this issue showing, for example, that brain regions critical to processing shifts in gaze are insensitive to violations of contextual cues in adult individuals with autism spectrum disorders (ASD; Pelphrey et al. 2005) as well as a lack of activity in fronto-parietal attentional networks in response to gaze cues in children with ASD (Greene et al. in press). Gaze processing abnormalities may be present early in development, and may underlie specific social deficits that emerge in autism, but the precise ways in which this might occur has incited great debate. The failure of children with autism to engage in normal, direct eye contact has led to the formulation of a “gaze aversion hypothesis” whereby these children are hypothesized to avoid mutual eye gaze because it is aversive or overly arousing to them, and some neuroimaging studies have highlighted neural mechanisms that may be involved (e.g., Dalton et al. 2005; see Bowman et al. 2004 for a discussion).

Alternatively, children with ASD may engage in reduced mutual eye contact or gaze monitoring because it may be intrinsically less interesting to them, and/or may not carry the same informational value as for TD children. This alternative model proposes reduced social motivation and cue salience, and suggests that this reduced salience, in turn, may negatively affect the development of expertise with social and emotional cues in children with ASD (Dawson et al. 1998; Klin et al. 2003). Neuroimaging studies by Schultz and others have offered partial support for such a hypothesis showing reduced activity in the region of the fusiform gyrus typically associated with face processing, a finding taken to reflect reduced social experience and face-processing specialization (Schultz et al. 2000; Grelotti et al. 2002; Pierce et al. 2001; Wang et al. 2004).

These two hypotheses make different predictions about brain activity during gaze and emotion processing. The former suggests that direct gaze, particularly in faces displaying strong affect, should produce hyperactivity in emotionally responsive brain regions, such as the amygdala and ventrolateral prefrontal cortex (VLPFC), areas known to be involved in emotion signaling, integration, and regulation (Bunge et al. 2002; Aron et al. 2004). The latter hypothesis predicts reduced responsiveness in these same neural systems to these stimuli. Previous studies have found reduced automaticity in recruiting social information processing regions such as the amygdala and frontal areas when presented with stimuli such as faces or voices (e.g., Dapretto et al. 2006; Wang et al. 2007). It is not clear, however, how eye gaze and emotion cues are integrated in the TD brain when processing emotional expressions with different gaze directions, nor how such cues, both important when navigating social interactions, may be abnormally processed in the autistic brain. Given their potential impact on early intervention, interpretation, and treatment of individuals with autism, we sought to compare the predictions of the above two hypotheses and build upon previous work on gaze and emotion processing in children with ASD, to help shed further light on the neural bases of these functions. More specifically, we performed fMRI during direct and averted gaze processing in children with ASD and TD controls to examine the impact of gaze direction on neural responses to social and emotional stimuli.

## Methods

### Participants

Sixteen TD children (two female) between the ages of 8–17 years (mean age 12.30) were gender-, age-, and IQ-matched to our sample of 16 children with ASD. For each child in the ASD group, a prior clinical diagnosis was confirmed in an initial lab visit using the Autism Diagnostic Interview, Revised (Lord et al. 1994) and Autism Diagnostic Observation Schedule-Generic (Lord et al. 2000) (see Table 1 for subject demographic information, and Supporting information for diagnostic details). In our sample, eight children met research criteria for diagnosis of autism on both the ADOS and ADI, five met diagnosis for autism by ADI and for ASD by the ADOS, two met diagnosis for ASD on both the ADOS and ADI, and one met diagnosis for ASD by the ADI and for autism by the ADOS. Prior to participation, all subjects and their parents provided written consent according to specifications by the Institutional Review Board at the University of California, Los Angeles.

**Table 1:** Subject demographics

	ASD	TD
Chronological Age (years ± SD)	11.69 ± 2.71	12.30 ± 1.88
Verbal IQ	100.38 ± 19.90	104.13 ± 17.43
Performance IQ*	111.13 ± 19.83	104.60 ± 12.69
Full Scale IQ	106.19 ± 20.31	105.60 ± 15.99
ADOS-G	12 ± 4.0	N/A
ADI-R	21.53 ± 7.7	N/A
Mean head movement during scan (mm)	.732 ± .742	.535 ± .434

* Represents a significant difference between groups. IQ domains assessed by the Wechsler Abbreviated Scale of Intelligence (WASI). Pearson 1999.

### Stimuli and materials

All children underwent an event-related fMRI session during which they viewed photographs of emotionally expressive faces (Tottenham et al. 2009) through magnet-compatible goggles. One hundred and sixty different faces depicted expressions of anger, fear, happiness, or a neutral expression, which for analyses purposes were classified as having either positive/neutral or negative valence. Half of the total faces displayed a direct gaze, and half displayed an averted gaze looking to the right or left of the observer. The gaze-averted images were produced by doctoring the eyes of the direct-gaze faces in Photoshop; therefore, gaze-averted and gaze-direct pairs of faces were identical in every respect apart from actors’ gaze direction.

### fMRI activation paradigm

Presentation of the stimuli comprised 20 trials for each of the eight conditions (angry, fearful, happy or neutral, each with direct and averted gaze) interspersed with null events. In the present study, we evaluated only the negative-valenced stimuli (i.e., angry and fearful expressions). Stimulus faces were presented in pseudo-random sequence for 2 sec each, yielding a run of 9 min in total. As children with ASD often have atypical gaze patterns, which may affect fMRI activation patterns (Dalton et al. 2005), we presented subjects with two cross-hair fixations prior to each stimulus. These were presented for 1 sec on a blank screen in the exact position where the eyes were to appear in the next face stimulus, in order to ensure that all subjects attended to the eye region. Null events consisted of fixation crosses in the center of a blank screen; these were distributed pseudo-randomly throughout the run and modeled as a separate condition. Each subject was presented with one of eight runs which had a different counterbalancing order of the experimental conditions. The presentation order of the individual stimuli was pseudo-randomized in a sequence designed to optimize statistical efficiency in the experimental design (Wager and Nichols et al. 2003). The order of the emotional expression and gaze conditions was counterbalanced between and within groups.

### Eye tracking

One possible confound in neuroimaging studies of face and gaze processing tasks in autism is the possibility that children with ASD may not actually look at the eyes (or look less at the eyes than TD controls). Our paradigm was designed to address this concern as fixation crosses were presented on the screen for 1 sec precisely in the region where the eyes of the next stimulus would appear. Although we were unable to track subjects’ eye movements during scanning, eye fixation in response to the identical paradigm was assessed in 21 of the original 32 children who participated in the study (10 TD and 11 ASD) during a separate eye tracking session conducted to test whether this manipulation was effective in driving attention to the eye region.

During this second visit, we tracked eye movements while each child sat 50 cm in front of a monitor, observing the identical sequence of faces as they saw previously in the scanner. Eye movements were calibrated for each subject and confirmed before and after the gaze data. Using an infrared Tobii 1750 eye tracking system (Tobii Technology), which calculates visual fixation within 1 cm of accuracy, we compared the amount of time subjects spent looking at the face and at the eyes, both in raw numbers and in percentage of total trial time spent fixating in the eye region. The results of these analyses indicate that the use of fixation crosses at the eye level was successful in drawing attention to the eye region during stimulus presentation as no significant differences were found between the groups in the amount of time spent looking at the eyes either during direct (*t* = 0.63, *P*>0.50) or indirect (*t* = 0.85, *P*>0.40) gaze, nor in the amount of overall looking time at the faces overall (all *P*-values >0.30).

### fMRI data acquisition

Imaging was performed using a 3T Siemens Allegra MRI scanner. For each subject, we acquired 270 interleaved functional T2*-weighted echoplanar images (EPI) [slice thickness, 3 mm/1mm gap; 36 axial slices covering whole brain volume; repetition time (TR), 2 sec; echo time (TE), 25 msec; flip angle, 90°; matrix, 64 × 64; field of view (FOV), 20 cm]. Two additional volumes were discarded at the beginning of each run to allow for T1 equilibrium effects. In addition, a T2-weighted matched-bandwidth high-resolution anatomical scan (same slice prescription as EPI) was acquired for each subject (TR: 5 sec; TE: 33 msec; matrix size: 128 × 128; FOV: 20 cm) for registration purposes into a Talairach-compatible MR atlas (Woods et al. 1999).

### fMRI data analysis

All functional images were registered using Automated Image Registration (AIR; Woods et al. 1998), whereby EPI images were first registered to the matched-bandwidth high-resolution structural image for a given subject and normalized into a Talairach-compatible MR atlas (Woods et al. 1999). Images were spatially smoothed using a 6 mm full-width half-maximum Gaussian kernel. Finally, for each subject, mean head motion was computed using AIR by averaging the displacements across all voxels in all functional images relative to their mean position during the scans (Woods et al. 2003), and it was confirmed that there were no differences in head motion between the groups.

Statistical analyses were performed using SPM99 (Wellcome Department of Cognitive Neurology, London, UK; http://www.fil.ion.ucl.ac.uk/spm). For each comparison of interest, we conducted within- and between-group random effects analyses using one- and two-sample *t*-tests, respectively, and defined statistical significance at a signal intensity magnitude of *P* < 0.01, and a corrected cluster size threshold corresponding to *P* < 0.05. All analyses reported were statistically corrected for multiple comparisons across the whole brain at the cluster level, unless otherwise noted.

## Results

### Within-group effects: TD controls

**Figure 1:**
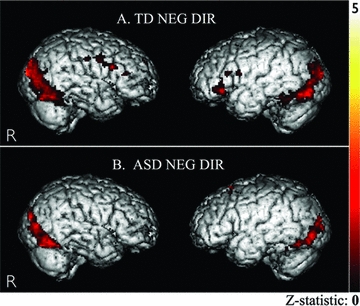
Negative direct. (A) TD group: BOLD signal changes while viewing negative-direct gaze (vs. null events) in bilateral visual-association cortices, bilateral VLPFC (BA 47), and right premotor cortex (BA 6). (B) ASD group: BOLD signal changes in bilateral visual-association cortices while viewing negative-direct gaze (vs. null events). For display purposes, images are thresholded at *t* > 2.60, *P* < 0.01, *k* > 20 voxels (uncorrected) although activity in these regions survived correction for multiple comparisons at the cluster level (see Table 2).

We first examined the effects of observing negative facial expressions with direct and averted gaze separately in comparison to null events for each group. When TD subjects viewed negative expressions with direct gazes (Fig. 1A), they recruited a network of regions associated with visual and face processing (e.g., occipital cortex and bilateral fusiform gyri). Also in response to direct gaze, they showed activation in frontal regions, including bilateral VLPFC extending into ventral inferior frontal gyrus on the left and premotor cortex, as well as in subcortical regions including bilateral amygdalae, left caudate head, and the pulvinar nucleus of the thalamus (Table 2).

**Table 2:** Peaks of activation while viewing faces with gaze-direct and gaze-averted negative expressions

			Direct - Null								Averted- Null							
				
			TD Group				ASD Group				TD Group				ASD Group			
						
Anatomical Region	BA	H	*x*	*y*	*z*	*Z*	*x*	*y*	*z*	*Z*	*x*	*y*	*z*	*Z*	*x*	*y*	*z*	*Z*
Premotor Cortex	6	R	54	−4	36	4.52												
IFG	45/47	L	−38	30	2	3.44												
IFG	47	L	−48	28	−6	3.55												
IFG	47	R	46	32	0	2.97												
Caudate (head)		L	−20	14	6	3.40*												
Thalamus, pulvinar n.		L	−22	−30	2	4.20												
Thalamus, pulvinar n.		R	18	−30	2	3.46												
Amygdala		L	16	−6	−8	3.01												
Amygdala		R	−20	−10	−6	2.74*												
Hippocampus/PHG		L													−26	−28	−2	3.69
Fusiform (medial)	37	L	−34	−50	−12	3.91	30	−60	−10	4.25	−38	−58	−8	3.40	34	−52	−12	3.90
Fusiform (medial)	37	R	34	−40	−16	4.60	24	−54	−10	3.91	38	−56	−10	3.79	24	−52	−8	3.88
Fusiform (lateral)	37	L	−44	−46	−12	3.82	−38	−52	−14	4.96	−40	−50	−14	2.97	−40	−50	−16	3.41
Fusiform (lateral)	37	R	32	−48	−10	4.38	34	−62	−12	4.67	32	−40	−18	3.45	34	−66	−12	4.88
Lingual Gyrus	17	L	−24	−84	2	4.79	0	−84	8	5.54	14	−88	8	5.08	0	−84	10	4.89
Lingual Gyrus	17	R	16	−86	2	4.86	16	−82	−2	4.70	−8	−92	8	4.52	−8	−86	2	4.37
MOG	18	L	−10	−80	0	6.04	−16	−78	−6	4.96	−12	−82	0	5.53	−18	−84	2	4.77
MOG	18	R	22	−84	−2	4.32	18	−72	−4	5.03	26	−66	4	4.23	6	−82	−2	5.11
Mid. Occip. Gyrus	19	L	−36	−80	−8	4.40	−14	−96	16	5.43	−28	−78	2	3.83	−18	−94	14	4.60
Mid. Occip. Gyrus	19	R	18	−94	18	5.98	6	−92	16	5.30	28	−84	24	4.65	22	−88	20	4.42

* Cluster survives correction for small volume.

*P* < 0.05, corrected for multiple comparisons, at the cluster level; *P* < 0.01, uncorrected for multiple comparisons, at the voxel level. Talairach coordinates.

BA = putative Brodmann Area; L and R = left and right hemispheres; *x*, *y*, and *z* refer to Talairach coordinates corresponding to left-right, anterior-posterior, and inferior-superior axes, respectively; *Z* refers to the highest *Z* score within a re

**Figure 2:**
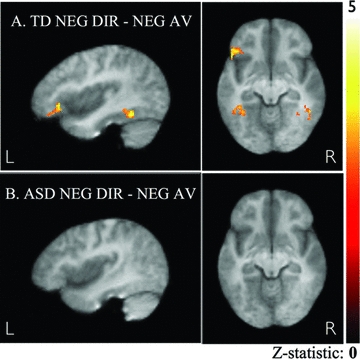
Negative direct–negative averted. (A) TD group: BOLD signal changes while viewing negative-direct versus negative-averted gaze in left VLPFC (*x*, *y*, *z* = −46, 28, −4, BA 47; 128 voxels). (B) ASD group: BOLD signal changes while viewing negative-direct versus negative-averted gaze. For display purposes, images are thresholded at *t* > 2.60, *P* < 0.01, *k* > 30 voxels (uncorrected); activity in the VLPFC cluster in the TD group survived correction for multiple comparisons at the cluster level.

In contrast, when TD children viewed these identical expressions in faces with averted gaze, we observed a striking difference in regional activation. While visual regions and fusiform gyri were almost identically activated, none of the areas active in gaze-direct conditions in frontal and prefrontal cortices, or in subcortical areas such as the amygdalae and caudate showed a statistically significant response relative to null events. A direct comparison of brain activity in response to gaze-direct versus gaze-averted negative emotion faces in the TD group (Fig. 2A) revealed left VLPFC (BA 47; *x*, *y*, *z* = −46, 28, −4; *Z* = 3.33), medial temporal gyrus (BA 37/21; *x*, *y*, *z* = 44, v60, 4; *Z* = 3.49), and fusiform gyrus (BA 37; *x*, *y*, *z* = −42, −50, −12; *Z* = 3.66) to be reliably more responsive to viewing direct as opposed to averted gaze (*P* < 0.05, corrected for multiple comparisons at the cluster level).

### Within-group effects: Children with ASD

When children with ASD viewed negative expressions with direct gazes (Fig. 1B and Table 2), as with the TD children, they too showed significant and extensive activation of occipital and fusiform cortices. These gaze-direct faces, however, elicited no significant signal changes in the inferior frontal or subcortical regions observed in the TD group, with activation limited to visual-association cortices (*P* < 0.05, corrected for multiple comparisons at the cluster level). An exploratory threshold (*P* < 0.05, uncorrected), revealed responses to negative-valence, direct gaze in left hippocampus, superior frontal gyrus, and medial parietal cortex, but we found no activity in VLPFC as had been observed in the TD group.

Also unlike the TD group, while viewing these same expressions with averted gaze, the ASD group showed a nearly identical pattern of activity as that in response to viewing gaze-direct conditions. A direct statistical comparison of brain responses of the ASD group to gaze-direct versus gaze-averted conditions showed no significant differences in activation (Fig. 2B).

### Between-group effects

**Figure 3:**
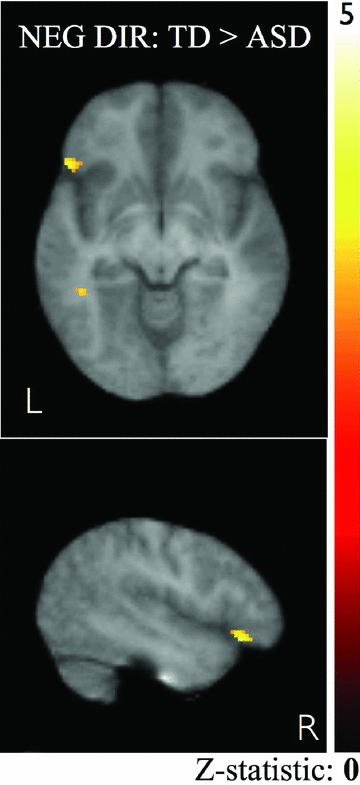
Negative direct–negative averted. TD > ASD: BOLD signal changes in left VLPFC (*x*, *y*, *z* = −50, 26, −8, BA 47; 47 voxels). For display purposes, images are thresholded at *t* > 2.60, *P* < 0.01, *k* > 20 voxels (uncorrected), with the reliable difference in VLPFC activity between groups surviving small volume correction for multiple comparisons at the cluster level.

To directly test the hypothesis that TD children showed selectively greater activation during direct-gaze processing of negative emotional faces compared to the ASD children, we contrasted brain responses to negative emotions versus null events between the groups, using both within-group results as a combined mask to restrict our search only within those regions that showed significant activity in either group. Viewing negatively valenced, gaze-direct faces elicited greater activation in the TD group in one region only: bilateral VLPFC (Fig. 3 and Table 3). In contrast, no region showed significantly more activation in the ASD than TD group for this gaze-direct contrast. For the gaze-averted contrasts, between-group differences were limited to a region in somatosensory cortex, which was significantly more active in the ASD group (Table 3). Finally, the between-group contrast assessing differences in response to gaze-direct versus gaze-averted images (i.e., the interaction effect between group and gaze condition) yielded a single cluster in left VLPFC (*P* < 0.05, corrected for small volume at the cluster level), confirming greater activity in this region in the TD versus the ASD group for direct versus averted eye gaze.

**Table 3:** Peaks of activation while viewing faces with negative emotions and direct or averted gazes, compared between TD and ASD groups

			Direct - Null				Averted -Null				Direct - Averted			
					
			TD > ASD				TD > ASD				TD > ASD			
					
Anatomical Region	BA	H	*x*	*y*	*z*	*Z*	*x*	*y*	*z*	*Z*	*x*	*y*	*z*	*Z*
VLPFC	47	L	−40	38	−2	3.64					−50	26	−8	3.69*
VLPFC	45	R	40	30	4	3.59								

			ASD > TD				ASD > TD				ASD > TD			
					
			x	y	z	*Z*	x	y	z	*Z*	x	y	z	*Z*
Somatosensory Cortex	2	L					−48	−22	46	3.30				

* Cluster survives correction for small volume. *P* < 0.05, corrected for multiple comparisons, at the cluster level; *P* < .01, uncorrected for multiple comparisons, at the voxel level. Talairach coordinates.

## Discussion

In the present study we found that TD children show marked regional increases in brain activity in response to negative emotional expressions conveying direct as opposed to averted gazes, where the facial expressions were otherwise identical. Sensitivity to this subtle stimulus alteration suggests that the significance of direct eye gaze in emotionally expressive faces is powerfully registered in the young brain during face processing. Interpreting and responding accordingly to whether or not cues conveyed about others’ mental or emotional states relate immediately to you or your actions is essential for successfully navigating a dynamic and complex social world. Processing direct gaze in faces displaying negative emotions generates a strong neural signature in the TD brain, marked by activity in a network of emotion-processing regions. The dynamic gaze-related component of face processing has been elegantly described and replicated in studies using moving eye stimuli, highlighting the importance of social context on neural response in both the adult and TD brain (Pelphrey et al. 2003, 2004; Mosconi et al. 2005).

Interestingly, brain activity in VLPFC in TD children was solely dependent on eye gaze direction in angry or fearful faces. VLPFC has been observed to respond during the labeling of negative emotions (Hariri et al. 2000), as well as while interpreting others’ mental or emotional states on the basis of these emotions (Sabbagh 2004), and is associated in both children and adults with enhanced cognitive control and suppression of undesired behavioral responses (e.g., Bunge et al. 2002; Aron et al. 2004). The relevance of gaze in processing the immediate threat and meaning of these negative emotional expressions suggests that differential activity in VLPFC may code or respond to the immediate, communicative significance of these emotional expressions. The results of this study suggest that in TD children, eye gaze cues may powerfully influence brain responses directly contributing to these interpretive and regulating functions within a social context.

The region in VLPFC differentiating direct and averted gaze in TD children also differentiated the TD from ASD group activation during direct gaze. Although children with ASD attended to the same visual information and fixated equally on the features of the face as did TD children (as confirmed in a separate eye tracking session), our data suggest that the particular significance of the emotional information conveyed by the faces with direct gaze may have been processed differently by TD children. A direct gaze conveying a strong, negative emotion has immediate significance for the individual, signaling potential threat and critical social information (i.e., I am in trouble; I have done something wrong; someone is angry at me, etc.). The same facial expression conveyed with an averted gaze changes the significance of that information, tagging it as less immediately relevant to the receiver. In our sample of TD children, VLPFC activation appears to occur not merely as a result of exposure to negative affect, but rather to negative affect that is perceived to be *directly relevant* to the individual. In autism, it appears that processing this information in others’ faces, likely relying in part on regions sensitive to gaze direction, is abnormal or absent, even when visual perception is clearly intact.

Activity in VLPFC has been found in previous studies to show an inverse relationship with activity in the amygdala in nonclinical samples while processing negative affect faces (Hariri et al. 2000 and Kim et al. 2004), supporting an emotional response regulation function of this region. We find activity in *both* VLPFC and the amygdala to be significantly reduced, however, while children with autism process fearful or angry faces, relative to typical levels, and that this difference is most pronounced in the processing of faces with direct gaze. Studies have reported heightened sensitivity to direct gaze in regions such as the amygdala and striatum in autism, supporting a gaze aversion hypothesis whereby individuals with autism avoid mutual gaze with others due to the overly arousing or aversive nature of such eye contact (e.g., Dalton et al. 2005). However, findings regarding responsiveness to these cues in the amygdala and purported arousal have been mixed. If individuals with ASD have reduced eye fixation due to hyperarousal to these cues, then we would predict that with equal amounts of eye fixation across groups, exposure to expressive faces with direct gaze in a group of ASD children should cause an *increased* response in the amygdala and other regions associated with anxiety and inhibitory regulation—not only relative to that in TD children, but also relative to response to the same faces with averted gaze. Our results do not support this hypothesis of anxiety-associated social aversion in autism. Rather, our results are more consistent with the reduced social motivation hypothesis (Dawson et al. 1998), in line with recent evidence indicating that social stimuli (e.g., a smiling face) fail to elicit activity in the reward system in children with ASD (Scott-Van Zealand et al. 2010). The present results extend this hypothesis by suggesting that children with ASD may engage in less-direct eye contact in part because they do not extract the communicative intent from direct gaze cues as do TD children, leaving the eyes no more informative or interesting than any other facial feature.

Our finding of reduced activity in VLPFC in the ASD group while viewing direct-gaze faces, despite equal engagement of visual cortex and fusiform gyrus, are consistent with other reports showing reduced spontaneous inferior-frontal and medial temporal lobe activity while children with ASD interpret others’ mental or emotional states (Wang et al. 2004). Our results are not likely explained by decreased fixation on the eyes or faces in the children with ASD, as indicated by a separate eye tracking session. It cannot be ruled out that differences in activation may have been related to decreased *perception or judgment* of gaze direction in the ASD group, as has been suggested by a recent study on gaze processing in individuals with autism (Ashwin et al. 2009). This possibility of reduced discriminative ability in ASD between direct and averted gaze, however, likely represents a related aspect of decreased sensitivity to gaze cues and their associated communicative significance, and thus might be expected given the findings of the current study.

An additional concern that emerges from comparing a clinical sample with a group of TD children is that the observed differences may be due to generally reduced brain response in the experimental group. This did not appear to be the case in our data, however, as the observed reductions in VLPFC, caudate, and other areas were regionally specific, with activity in other visual- and face-processing regions found to be comparable between groups. Additionally, the children with autism in our study recruited other brain regions to a greater degree than TD children while viewing faces with averted gaze. At even the highest thresholds explored, significantly increased activity relative to that in the TD group was observed within somatosensory cortex (BA 2). As our paradigm encouraged each group to fixate on the eyes, these fMRI findings of somatosensory cortical activation in the ASD group are consistent with data from previous fMRI and eye tracking studies suggesting that children with ASD, unless otherwise instructed, may spontaneously use alternative strategies to process or interpret information in faces (e.g., Klin et al. 2002; Pelphrey et al. 2002; Wang et al. 2004; Dapretto et al. 2006; Wang et al. 2007). Further investigations of the fixation behavior of children with autism while viewing faces not only of varying emotions but also of varying eye gaze may be fruitful in identifying these potentially unique strategies. Furthermore, employing eye and emotion-related dynamic facial stimuli rather than stationary faces, as in the present study, may enrich our preliminary understanding of how dynamic gaze and emotion cues may modulate one another in the brain (Pelphrey et al. 2007).

The findings of our study are also in line with other data reporting decreased frontal brain activity in children with autism to emotional and social cues, suggesting that children who develop autism may have reduced integrity of frontal-posterior brain connections (Just et al. 2004, 2007). Several fMRI studies in autism have reported reduced left IFG activity in response to social cues, and both functional and structural data have supported a dysregulation model, whereby desynchronized and reduced prefrontal response during social tasks are results of distally reduced, and possibly locally increased, cortical connectivity (Courchesne et al. 2001; Herbert et al. 2004; Just et al. 2004, 2007). The results of our study are consistent with this theoretical explanation, but cannot directly address it.

Our experimental set-up with cross-hair fixation points preceding eye stimuli was designed to prevent gaze aversion or reduced fixation on the eyes in the ASD group, and our eye tracking data showed no group differences in gaze behavior in either gaze direction condition, making it unlikely that gaze aversion could have explained our results. Equivalent activation among ASD and TD children in visual-processing regions including the fusiform gyrus, which is critical for processing faces, further suggests that ASD and TD children spent equal time looking at the faces. Our inability to track eye fixation in the scanner during the fMRI sessions, however, represents a weakness of this study, which our separate eye tracking data can only indirectly address. Based on the eye-tracking findings, the fixation cross manipulation in our design may have helped equate fixation behavior between groups, as might have the fact that the ASD group represented a relatively high-functioning sample of children who, even without the fixation crosses, may not have demonstrated as dramatic fixation deviations as has been found in lower-functioning samples (Boucher and Lewis 1992).

We found that the amount of time that children tended to fixate on the face or particular regions of the face (as measured in the separate eye tracking session) did not relate in either group to brain activity in the amygdala, right VLPFC, or left VLPFC. Children with ASD who tended to look more at the eyes during direct gaze faces as a *proportion of time spent looking at other regions* such as the nose or forehead, however, did show significantly increased activation in right VLPFC during the presentation of negative, direct-gaze expressions. The presence of this relationship when eye gaze is quantified as a fixation preference, but not when it is quantified in terms of raw time, points to the possibility that children with a more normative bias to attend to eyes also show more normative brain activity. Children who overall attended to the faces less, but gazed more exclusively at the eyes when doing so, or children who attended well to the faces but showed a more distributed pattern of fixation did not show this associated increase in activation in VLPFC.

As the first study to directly address how gaze may be processed along with emotional content in TD children and children with autism, our results suggest that high-functioning children with ASD may perceive the faces and gaze direction, but that this information may not be automatically translated into its communicative significance through the co-recruitment of prefrontal and limbic brain regions, as appears to occur in children without ASD. If this is the case, deficits in social comprehension and functioning may not result directly from avoiding the eyes, or having a physiological aversion to direct gaze, but rather because the significance of emotional expressions with direct gaze are not extracted from their corresponding facial cues. This would suggest that at least by later childhood, reduced mutual gaze might be due to the fact that observing direct gaze in another person is no more meaningful or rewarding than observing a gaze that is averted. The differences we report between neurotypical children and children with ASD who display marked social impairments highlight the importance of appropriate sensitivity to the eye gaze in navigating the social world and suggest that disordered development in ASD may directly result from failure to appropriately respond to these subtle social cues.
